# Retention rate of biologic and targeted synthetic anti-rheumatic drugs in elderly rheumatoid arthritis patients: data from GISEA registry

**DOI:** 10.3389/fmed.2024.1349533

**Published:** 2024-03-11

**Authors:** Andreina Manfredi, Marco Fornaro, Chiara Bazzani, Simone Perniola, Alberto Cauli, Alessandra Rai, Ennio Giulio Favalli, Serena Bugatti, Maurizio Rossini, Rosario Foti, Fabrizio Conti, Giuseppe Lopalco, Anna Scalvini, Cristina Garufi, Mattia Congia, Roberto Gorla, Elisa Gremese, Fabiola Atzeni, Roberto Caporali, Florenzo Iannone, Marco Sebastiani

**Affiliations:** ^1^Rheumatology Unit, Azienda Ospedaliera Policlinico di Modena, University of Modena and Reggio Emilia, Modena, Italy; ^2^Rheumatology Unit, Department of Precision and Regenerative Medicine and Jonic (DiMePRe-J), University of Bari, Bari, Italy; ^3^Rheumatology and Clinical Immunology, ASST Spedali Civili, Brescia, Italy; ^4^Clinical Immunology Unit, Department of Geriatrics, Orthopedics and Rheumatology, Fondazione Policlinico Universitario A. Gemelli-IRCCS, Catholic University of the Sacred Heart, Rome, Italy; ^5^Rheumatology Unit, Azienda Ospedaliero-Universitaria di Cagliari, University of Cagliari Monserrato, Cagliari, Italy; ^6^Department of Rheumatology and Medical Sciences, ASST Gaetano Pini-CTO, Milan, Italy; ^7^Department of Clinical Sciences and Community Health, University of Milan, Milan, Italy; ^8^Department of Internal Medicine and Therapeutics, Università di Pavia, Pavia, Italy; ^9^Division of Rheumatology, Fondazione IRCCS Policlinico San Matteo, Pavia, Italy; ^10^Rheumatology Unit, University of Verona, Verona, Italy; ^11^Rheumatology Unit, San Marco Hospital, Policlinico University of Catania, Catania, Italy; ^12^Dipartimento di Scienze Cliniche Internistiche, Anestesiologiche e Cardiovascolari – Rheumatology Unit, Sapienza Università di Roma, Rome, Italy; ^13^Rheumatology Unit, Department of Clinical and Experimental Medicine, University of Messina, Messina, Italy

**Keywords:** rheumatoid arthritis, elderly, comorbidities, treatment, retention rate, safety

## Abstract

**Objectives:**

An increased number of elderly individuals affected by rheumatoid arthritis (RA) has been reported, including both patients with RA onset in advanced age and patients aged with the disease. In this registry-based study, we aimed to analyze the retention rate and cause of discontinuation of biologic (b) and targeted synthetic (ts)-disease-modifying anti-rheumatic drugs (DMARDs) in RA patients over 65 year old.

**Methods:**

RA patients enrolled in the Italian GISEA registry and starting a b- or a ts-DMARD over 65 years of age were included. Demographic, clinical, serologic, and therapeutic features were collected.

**Results:**

A total of 1,221 elderly RA patients were analyzed (mean age 71.6 ± 5.2 years). RA was diagnosed before 65 years in 72.5% of cases, a 60.6% of patients experienced a previous b- or ts-DMARD. In patients older than 65 initiating a new b- or ts-DMARDS, tumor necrosis factor alpha inhibitors (TNFi) were prescribed in 29.6% of patients, abatacept in 24.8%, anti-interleukin 6 receptor antagonists (anti-IL6R) in 16.3%, Janus kinases inhibitors (JAKi) in 24.9%, and rituximab in 4.4%. The main causes of discontinuation were primary or secondary inadequate responses (66.1%). The median retention rate for all treatments was 181.3 weeks. A statistically higher retention rate was observed for abatacept when compared to TNFi (*p* = 0.02), JAKi (*p* < 0.001), and anti-IL6R (p < 0.001), and for TNFi vs. JAKi (*p* = 0.013).

**Conclusion:**

We described, in a real-life setting, elderly RA patients treated with a biologic or a ts-DMARD in Italy. Loss of efficacy was the main cause of discontinuation, and the DMARD safety profile suggests that age does not contraindicate their use. Our study reinforced that the control of disease activity is mandatory.

## Highlights

- Loss of efficacy is the main cause of drug discontinuation in elderly rheumatoid arthritis patients.
- Comorbidities and disease activity are associated with drug discontinuation in elderly rheumatoid arthritis patients.
- In elderly RA patients, abatacept and TNF-alpha inhibitors are associated with a better retention rate.

## Introduction

Over the past years, an increased number of elderly individuals affected by rheumatoid arthritis (RA) has been observed (1, 2). In this group, both patients with RA onset at an advanced age and patients with an early occurrence of RA aged with the disease were included.

The increased age of the RA population can be attributed primarily to the improved management of the disease and associated conditions, as well as the overall increase in life expectancy (3, 4).

Among elderly RA patients, those with late-onset disease are described as experiencing more severe joint involvement and systemic symptoms; however, existing literature indicates that rheumatologists tend to adopt a less aggressive treatment approach in elderly patients (5). In this population, rheumatologists often postpone the use of biologic drugs, instead opting for prolonged use of steroids or non-steroidal anti-inflammatory drugs (NSAIDs) and employing a less intensive “treat to target” strategy (5).

Several factors contribute to the complexity of treating RA in elderly patients, such as the presence of other medical conditions, the use of multiple medications (polytherapy), extra-articular manifestations of RA, and the aging of the immune system (immune-senescence), which can lead to increased frailty (1).

Currently, treatment guidelines do not take into account the age or comorbidities of RA patients, and clinical trials typically focus more on younger individuals with RA (6).

The “Gruppo Italiano per lo Studio della Early Arthritis” (Italian Group for the Study of Early Arthritis; GISEA) includes 21 hospital and community-based rheumatology units throughout Italy. It has developed and maintained a nationwide registry to promote the study of patients with inflammatory arthritis according to standard-of-care criteria (7, 8).

The main objective of this study was to analyze the rheumatologists’ preferences in the prescription of biologic (b-) and targeted synthetic (ts)-disease-modifying anti-rheumatic drugs (DMARDs) in RA patients aged at least 65 years. Additionally, our study aimed to investigate the retention rate and the cause of discontinuation of biologics and ts-DMARDs in the elderly RA population enrolled in the GISEA registry.

## Patients and methods

Among subjects included in the GISEA registry, patients were enrolled in the study when a biologic (b- or ts-DMARD) was started over 65 years of age in a period between 1 January 2019 and 31 December 2021, independently from previous therapies with b- or ts-DMARDs. For patients treated with more b- or ts-DMARDs prescribed after 65 years of age, only the first was evaluated.

The GISEA registry enrolled patients classified as affected by RA according to the 1987 or 2010 American College of Rheumatology (ACR) criteria (2). Data evaluated for the study included age, gender, disease duration, use of oral corticosteroids (CSs), and conventional DMARDs (namely, methotrexate, leflunomide, sulphasalazine, and hydroxychloroquine), body mass index (BMI), presence and number of comorbidities, presence and number of extra-articular RA manifestations, 28-joint Disease Activity Score (DAS28) and clinical disease activity index (CDAI), C-reactive protein (CRP) and erythrocyte sedimentation rate (ESR), anti-citrullinated peptide antibodies (ACPAs), and rheumatoid factor (RF). Comorbidities recorded included anemia, anxiety/depression, cardiovascular diseases (coronary artery diseases, chronic heart failure, and arrhythmias), arterial hypertension, cerebrovascular diseases, liver diseases, acute and chronic kidney diseases, peripheral vasculopathy, diabetes, chronic obstructive pulmonary diseases, osteoporosis, thyroid dysfunction, and cancer. RA-related extra-articular manifestations (rheumatoid nodules, interstitial lung disease, Sicca syndrome, and vasculitis) were also collected. Causes of drug discontinuation (inadequate response, side effects, remission, or other) were recorded. We defined inadequate response as failure in the achievement of a remission or a low disease activity according to EULAR recommendations (9). A secondary inadequate response was defined as a failure of the treatment after a first good response (9). Patients with more than 15% of the missing data were excluded from the analysis. The study was approved by the local ethical committee of “Area Vasta Emilia Nord,” and each patient gave their consent.

### Statistical analysis

Continuous variables have been reported as mean ± standard deviation, median and interquartile (IQR) range, and categorical variables were reported as absolute numbers or percentages. Differences among patients who discontinued or continued treatment were analyzed using the Mann–Whitney test for non-parametric variables, and chi-square or Fisher tests, when appropriate, were used for categorical variables. Global persistence in therapy and the 2-year retention rate were evaluated by the mean of Cox regression. Then, a multivariate analysis was performed to analyze the effect of features at baseline on patients with regard to drug discontinuation (10). Analyses were performed using the STATA14 software (StataCorp LLC, College Station, TX, USA), with a value of p of ≤0.05 considered to be statistically significant.

## Results

We analyzed data from 1,221 elderly patients affected by RA; the female-to-male ratio was 3.79, and the mean age was 71.6 ± 5.2 years (23.7% of patients were older than 75); 55.3% of patients were positive for both RF and ACPA, while 77.8% of patients showed at least one of them. RA was diagnosed before 65 years in 72.5% of patients, while the diagnosis was performed over 65 years in 27.5% of patients. The mean disease duration was 14.6 ± 10.4 years. Comorbidities were detected in 55.6% of the population. A previous treatment with one or more b-DMARDs was discontinued in 60.6% of patients.

The demographic, clinical, and serological features of the patients enrolled are detailed in Table 1.

**Table 1:** Demographic, clinical, and serological features of 1,221 elderly rheumatoid arthritis patients enrolled in the study.

Patients enrolled	1,221
Females/Males (number)	966/255
Age at enrollment (mean ± SD)	71.6 ± 5.2
Disease duration, years (mean ± SD)	14.6 ± 10.4
Age at diagnosis ≥65 years	27.5
ACPA (%)	66.1
Rheumatoid factor (%)	67.0
Comorbidities (%)	55.6
BMI (mean ± SD)	25.7 ± 4.8
First-line bDMARD (%)	39.4
Steroid therapy (%)	57.9
csDMARDs (%)	58.6
Methotrexate (%)	46.7
ERS (mmh; mean ± SD)	31.9 ± 23.1
C-Reactive Protein (mg/L; mean ± SD)	23.6 ± 19.9
Tender joints 28 (mean ± SD)	4.7 ± 5.4
Swollen joints 28 (mean ± SD)	2.7 ± 4.0
CDAI (mean ± SD)	16.5 ± 4.3
DAS28 (mean ± SD)	4.0 ± 1.4

Continuous values are reported as mean and standard deviation (SD); dichotomic values are reported as percentage; ACPA, anti-citrullinated peptide antibody; RF, rheumatoid factor; DMARDs, disease-modifying anti-rheumatic drugs; ERS, erythrosedimentation rate; DAS28, disease activity score on 28 joints; CDAI, clinical disease activity index; BMI: body mass index.

Among patients enrolled, the first b- or ts-DMARDs prescribed over 65 years of age were a tumor necrosis factor-alpha inhibitor (TNFi) in 362 (29.6%) patients, and abatacept (CTLA4-Ig) in 303 (24.8%); 199 patients (16.3%) used anti-interleukin 6 receptor antagonists (anti-IL6R): Janus kinase inhibitors (JAKi) were the therapy chosen for 304 patients (24.9%), while rituximab (RTX) was prescribed in 53 patients (4.4%). Among the specific drugs, abatacept was the most prescribed drug (303 patients, 24.8% of the prescriptions), followed by baricitinib (198, 16.2%) and etanercept (186, 15.2%) (Table 2). The b- or ts-DMARD was prescribed as first-line after MTX in 39.4% of cases.

**Table 2:** Biologic or targeted synthetic DMARDs in 1221 elderly patients.

	N	%	Female/Male	First-line	cDMARDs	Glucocorticoids
TNF-alpha inhibitors	362	29.6	281/81	42%	63.5%	47.2%
Etanercept	186	51.4	143/43	47.8	62.4%	45.2%
Adalimumab	100	27.6	75/25	44%	68.0%	50.0%
Certolizumab	37	10.2	31/6	24.3%	62.2%	51.4%
Golimumab	29	8.0	25/4	27.6%	51.7%	48.3%
Infliximab	10	2.8	7/3	20%	80.0%	40.0%
Abatacept	303	24.8	235/68	45.9%	76.2%	67.0%
Anti-IL6R	199	16.3	151/48	37.7%	49.2%	58.3%
Tocilizumab	157	78.9	120/37	35.7%	48.4%	56.7%
Sarilumab	42	21.1	31/11	45.2%	52.4%	64.3%
JAK inhibitors	304	24.9	253/51	33.6%	41.8%	61.2%
Baricitinib	198	65.1	162/36	33.3%	41.4%	60.6%
Tofacitinib	83	27.3	73/10	36.1%	44.6%	63.9%
Upadacitinib	19	6.25	14/5	21.1%	31.6%	57.9%
Filgotinib	4	1.3	4/0	50%	100%	50.0%
Rituximab	53	4.4	46/7	24.5%	54.7%	58.5%

DMARDs, disease-modifying anti-rheumatic drugs; anti-IL6R, anti-interleukin 6 receptor.

Data about age, sex, combination therapy with conventional synthetic DMARDs (csDMARDs), and GC for each biologic or ts-DMARDs class are summarized in Table 2.

On average, patients had 1.35 ± 1.6 comorbidities each. Comorbidities were 1.6 ± 1.9 for patients treated with RTX, 1.5 ± 1.6 for CTLA4-Ig, 1.4 ± 1.7 for anti-IL6, 1.3 ± 1.5 for TNFi, and 1.2 ± 1.5 for JAKi, respectively.

### Median drug persistence

During a median follow-up of 61 weeks (IQR 26.4–104), 409 (33.5%) patients discontinued their treatment, 117 (38.5%) with JAKi, 115 (31.8%) with TNFi, 81 with both anti-IL6R (40.7%), 76 with CTLA4-Ig (25.1%), and 20 (37.7%) with RTX.

**Figure 1:**
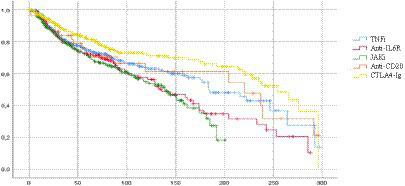
Cumulative survival for each class of biologic or targeted synthetic DMARDs in elderly patients. Median drug persistence for all treatments was 181.3 weeks (CI 95% 157.7–204.9). Significant differences in mean survival were observed between the different classes of drugs for CTLA4-Ig vs. TNFi (*p* = 0.02), CTLA4-Ig vs. JAKi (*p* < 0.001), CTLA4-Ig vs. anti-IL6 (*p* < 0.001), and TNFi vs. JAKi (*p* = 0.013). TNFi, TNF-alfa inhibitors; anti-ILR, anti-interleukin 6 receptor inhibitors; JAKi, Janus kinase inhibitors; anti-CD20, rituximab; CTLA4-Ig, abatacept.

The median retention rate for all treatments was 181.3 weeks (CI 95% 157.7–204.9). In details, median retention rates for each class of drugs were: 254.1 weeks for CTLA4-Ig (CI 95% 219.4–288.9), 220.7 for RTX (CI 95% 104.2–337.2), 184 for TNFi (CI 95% 151.1–216.8), 136 for anti-IL6R (CI 95% 105.1–166.9), and 139.1 for JAKi (CI 95% 114.7–163.6). A statistically higher retention rate was observed at Mantel–Cox analysis for CTLA4-Ig when compared to TNFi (*p* = 0.02), JAKi (*p* < 0.001), and anti-IL6R (*p* < 0.001), and for TNFi vs. JAKi (*p* = 0.013) (Figure 1).

At 2 years, the cumulative retention rate from the beginning of the treatment was 54.9% ± 3.7; in particular, the 2-year retention rate was 66.3% ± 3.8 for CTLA4-Ig, 59.7% ± 8.8 for anti-CD20, 54.9% ± 3.7 for TNFi, 49.2% ± 0.5 for anti-IL6R, and 52.7% ± 3.7 for JAKi.

### Predictive factors of discontinuation of all treatments

At univariate analysis, comorbidities, male sex, high disease activity (calculated with DAS28 and CDAI), a low BMI, and the second or third line of b- or ts-DMARDs therapy were associated with drug discontinuation. No differences were observed after stratification according to age at diagnosis. A combination therapy with MTX or other DMARDs reduced the risk of drug discontinuation. Among classes of b- or ts-DMARDs, treatment with JAKi or anti-IL6R drugs was associated with reduced persistence in therapy. On the contrary, CTLA4-Ig was associated with a lower risk of drug discontinuation (Table 3).

**Table 3:** Association between demographic, clinical, and therapeutic features and drug discontinuation in 1221 elderly patients (univariate analysis).

	Hazard ratio	95% Confidence interval		*p*
Age at diagnosis	0.998	0.989	1.008	0.719
Age at enrollment	0.985	0.965	1.004	0.125
Disease duration (months)	1	0.999	1.001	0.981
Male sex	1.341	1.047	1.718	0.017
CDAI	1.022	1.013	1.032	<0.001
RF	0.98	0.77	1.26	0.87
ACPA	0.95	0.75	1.21	0.7
RF or ACPA	0.96	0.9	1.03	0.23
Line of therapy (b- or ts-DMARDs)	1.098	1.039	1.159	0.001
CTLA4-Ig	0.903	0.858	0.949	<0.001
TNFi	0.943	0.760	1.171	0.597
JAKi	1.132	1.052	1.218	0.001
anti-IL6R	1.135	1.005	1.282	0.047
RTX	0.976	0.871	1.093	0.674
MTX	0.813	0.668	0.989	0.039
Oral corticosteroids	1.207	0.987	1.476	0.065
DMARDs	0.801	0.659	0.974	0.026
Presence of comorbidities	1.276	1.047	1.555	0.016
BMI	0.965	0.938	0.993	0.014

CDAI, clinical disease activity index; RF, rheumatoid factor; ACPA, anti-citrullinated peptide antibody; bDMARDs, biologic disease-modifying anti-rheumatic drugs; tsDMARDs, targeted synthetic DMARDs; CTLA4-Ig, Abatacept; TNFi, TNF-alfa inhibitors; JAKi, Janus kinases inhibitors; anti-IL6R, anti-interleukin 6 receptor; RTX, rituximab; MTX, methotrexate; DMARDs, disease-modifying anti-rheumatic drugs; BMI, body mass index.

CDAI (HR 1.022, CI 95% 1.012–1.032, *p* < 0.001) and comorbidities (HR 1.331, CI 95% 1.035–1.711, p = 0.026) were independently associated with drug discontinuation, while CTLA4-Ig (HR 0.683, CI 95% 0.477–0.977, *p* = 0.037) was associated with a longer persistence in therapy in a Cox multivariate analysis model, including CDAI, sex, CTLA4-Ig, therapy with anti-IL6R or JAKi, combination therapy with a csDMARD, comorbidities, and line of b- or ts-DMARDs.

### Predictive factors of discontinuation of treatment for each class of drugs

For each class of drugs, univariate analysis was performed to evaluate possible predictive factors for discontinuation. No findings were associated with an increased discontinuation rate for anti-IL6R, while multivariate analysis showed a RA diagnosis over 65 years as independent factors associated with discontinuation for JAKi, the number of comorbidities for RTX, and a high CDAI for TNFi. For CTLA4-Ig, a BMI < 20, older age, and a non-first line of treatment were associated with increased drug discontinuation. Combination therapies with glucocorticoids or csDMARDs did not significantly change the retention rates of ts- and b-DMARDs (Table 4).

**Table 4:** Factors associated with drug discontinuation according to a class of biologic or targeted synthetic DMARDs (multivariate analysis).

	Odds ratio	95% Confidence interval		*p*
Abatacept				
Male sex	1.20	0.57	2.52	0.64
Age	1.09	1.02	1.15	0.007
BMI < 20	2.69	1.51	5.87	0.013
No first-line treatment	2.98	1.51	5.88	0.002
IL6- inhibitors				
----	----	----	----	----
JAKi				
CDAI	1.02	1.00	1.04	0.036
Male sex	1.03	0.53	1.99	0.93
Diagnosis >65 years old	0.58	0.32	1.07	0.08
No first-line treatment	1.36	0.77	2.42	0.29
Rituximab				
Age	0.97	0.87	1.06	0.43
Male sex	2.14	0.49	9.28	0.31
Comorbidities	2.66	1.06	6.69	0.037
TNFi				
Male sex	1.44	0.75	2.78	0.27
CDAI	1.04	1.03	1.06	<0.001
Diagnosis >65 years old	1.21	0.69	2.11	0.51
No first-line treatment	0.87	0.49	1.54	0.64

DMARDs, disease-modifying anti-rheumatic drugs; BMI, body mass index; JAKi, Janus kinases inhibitors; TNFi, TNF-alfa inhibitors; CDAI, clinical disease activity index.

### Causes of discontinuation of biologic/synthetic drugs

**Figure 2:**
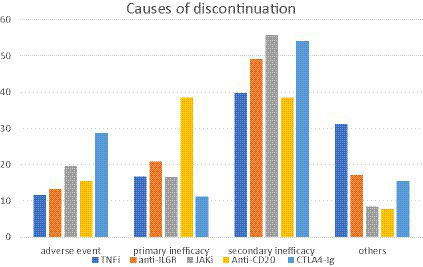
Main causes of discontinuation for each class of drugs. Among 1,221 patients evaluated, 33.5% discontinued treatment. Considering all the entire population, the main causes of discontinuation were adverse events (18.1%), primary or secondary inadequate responses (17.1 and 49.3%, respectively), and others (15.4%). TNFi, TNF-alfa inhibitors; anti-ILR, anti-interleukin 6 receptor inhibitors; JAKi, Janus kinase inhibitors; anti-CD20, rituximab; CTLA4-Ig, abatacept.

Among the 1,221 patients evaluated, 33.5% discontinued treatment. Discontinuation was observed in 31.5% of patients treated with TNFi, 40.7% of patients treated with anti-IL6R, 38.5% of patients treated with JAKi, 37.7% of patients with anti-CD20, and 25.1% of patients treated with CTLA4-Ig. Considering the entire population, the main causes of discontinuation were adverse events (18.1%), primary or secondary inadequate responses (17.1 and 49.3%, respectively), and others (15.4%). The main causes of discontinuation for each class of drugs are summarized in Figure 2.

## Discussion

In the last few years, the number of RA patients older than 65 has significantly increased, accounting for more than 30% of the RA population (1, 3, 5, 7). This observation refers to both patients with RA onset at an advanced age, and patients with an early occurrence of the disease. Elderly onset RA is generally associated with a more balanced gender distribution, a higher frequency of acute onset, and more frequent involvement of large joints, sometimes complicating differential diagnosis with polymyalgia rheumatica (11, 12). Although a more aggressive evolution of RA has been described in elderly patients, existing data reveal that conventional and biologic DMARDs are under-used in elderly RA patients (1, 5, 13).

Since elderly patients are frequently excluded from clinical trials, evidence-based data are scarce to develop guidelines for this population (6). For this reason, the pathophysiological aspects of aging and their implications in the treatment of RA in older patients need to be further investigated, as these aspects not only influence the clinical manifestations of RA onset but also impact the therapeutic options that can be proposed in this category of patients (1, 3, 4, 6, 14).

The large number of subjects included in the GISEA registry allowed us to describe, in a real-life setting, the features of elderly RA patients in Italy treated with a biologic or a ts-DMARD.

A few studies have specifically compared the retention rates of biologic DMARDs in elderly patients (15, 16), and then we aimed to evaluate possible differences in the persistence in therapy of different biologic and ts-DMARDs in patients older than 65 years and to investigate possible predictive factors for treatment failure for each drug.

Abatacept was the first b-DMARD prescribed (24.8% of the total) in our study. Despite the availability of cheaper biosimilar drugs, such as adalimumab and etanercept, abatacept was prescribed as a first-line biologic drug after conventional DMARDs in 45.9% of cases, suggesting that Italian rheumatologists might perceive abatacept as the safest option for elderly patients. Baricitinib and tofacitinib, more recently introduced in Italy, have also been prescribed to a large number of patients, similar to TNFi. On the contrary, RTX has been prescribed only to a small number of patients. In this regard, the pandemic could have modified the therapeutic approach of Italian rheumatologists because of both the reduced access of elderly patients to hospitals and the potential negative effect of RTX on the response to the anti-SARS-CoV-2 vaccination (17–19).

Like for younger patients (20, 21), an insufficient clinical response remains the first cause of drug discontinuation in this specific population (22). Approximately half of patients showed a secondary loss of drug response. Clinical disease activity measured with CDAI and the number of comorbidities were the main factors associated with drug discontinuation. Of interest, combination therapy with a csDMARD, mainly MTX, was associated with longer persistence on therapy, but the association was not confirmed by multivariate analysis. This point could be relevant in elderly patients, in whom comorbidities and multi-therapies can increase the risk of side effects (1, 23). In this regard, monotherapy might be a driver of choice for JAKi and anti-IL6R, combined with a csDMARD, in less than half of patients.

Bechman et al. evaluated the survival rates of TNFi in combination therapy with MTX or not in RA patients older than 75 years. In biologic naïve patients ≥75-years-old starting with TNFi, the authors did not observe an increased risk of discontinuation in patients treated with monotherapy (differently by younger patients included in the study). In elderly patients, this may be related to the phenomenon of immunesenescence, which induces a lower production of anti-drug antibodies (24, 25).

Specker et al. observed good safety and efficacy of tocilizumab (anti-IL6R) in RA patients >65 years old at baseline. Patients >65 years had higher disease activity scores, lower physical functioning, and a greater number of comorbidities at baseline. However, they had numerically greater reductions of CDAI over the course of treatment than patients <50 years. Furthermore, the presence of comorbidities did not modify the results of disease activity improvement (26).

Regarding abatacept, a small retrospective Japanese study reported no significant differences in the incidence of adverse events between younger and elderly RA patients (≥75 years), while the overall retention rate of CTLA4-Ig and infection-free survival were similar in the two groups. Finally, the most common cause of discontinuation was treatment inefficacy, independently of age (27, 28). Similar results were also described by Temmoku et al. in patients treated with a JAKi. No differences were reported in retention rate and causes of drug discontinuation in old (≥ 65 years) or very old (≥ 75) RA patients who initiated a JAKi (29).

In elderly patients from the ANSWER cohort study, abatacept and tocilizumab showed the highest retention rates, 78.1 and 66.8% at 36 months, respectively, and both showed a higher retention rate compared with TNFi (16).

Differently, in our study, abatacept was the drug with the best persistence in therapy (254.1 weeks, CI 95% 219.4–288.9) and a retention rate at 2 years of 66.3% ± 3.8, but persistence in therapy of tocilizumab was similar to TNFi and significantly lower than abatacept.

A specific point to be addressed in elderly patients is the relevance of body weight in response to the therapy. In young patients, BMI is associated with lower effectiveness for many drugs (30, 31).

On the contrary, in our study, BMI was inversely associated with drug discontinuation. In elderly patients, maintenance of body weight is usually correlated with their health status, and a low BMI might be associated with sarcopenia (32). To reinforce this hypothesis, a very low BMI (< 20) is associated with a high discontinuation rate in elderly RA patients treated with CTLA4-Ig.

Recently, the results of the Oral Surveillance trial suggested an association between tofacitinib and cardiovascular adverse events and malignancies rather than TNFi in patients with RA (33). Consequently, the European Medicine Agency (EMA) and the Food and Drug Administration modified the labeling of JAKi. Our data were collected before the labeling revision from EMA. Our results did not suggest any difference in safety between JAKi and different classes of b-DMARDs. In this regard, a careful evaluation of cardiovascular risk is mandatory for over 65 patients.

Our data were collected before the labeling change, and only future studies might clarify its possible impact on the prescription of JAKi in over 65 patients.

Finally, for each class of drugs, different factors associated with early discontinuation were identified. Despite the fact that we are far from true personalized therapy, some drivers of choice might be identified, suggesting a possible therapeutic algorithm according to the clinical features of the disease. For example, according to our results, abatacept and anti-IL-6R should be preferred in patients with high CDAI at baseline.

Further, the safety profile observed in this study suggests that the age of patients does not contraindicate the use of these drugs, reinforcing the opportunity to control disease activity in this group of patients.

Our study has some limitations; in fact, comorbidities are reported as “number of comorbidities” and cannot be further detailed. Therefore, we cannot advance any suggestion about the relative role of specific organ involvement (namely cardiovascular and pulmonary) on drug discontinuation. Moreover, smoking habits were not available for our analysis. On the other side, the current is one of the largest studies on elderly RA patients, allowing for the first time to compare the retention rates of biologics and ts-DMARDs in patients over 65 years old.

In conclusion, despite some limitations, our study allows us to provide an accurate picture of the treatment of elderly RA patients, which should be evaluated according to specific features such as comorbidities and age at diagnosis. While waiting for a clinical trial designed for this group of patients, registries could help us identify the best therapeutic approach and the effect of specific comorbidities on drug efficacy and safety in elderly RA patients.

## Data availability statement

The raw data supporting the conclusions of this article will be made available by the authors, without undue reservation.

## Author contributions

AM: Writing – original draft, Writing – review & editing. MF: Validation, Writing – review & editing. CB: Validation, Writing – review & editing. SP: Validation, Writing – review & editing. AC: Validation, Writing – review & editing. AR: Writing – review & editing, Writing – original draft. EF: Validation, Writing – review & editing. SB: Validation, Writing – review & editing. MR: Validation, Writing – review & editing. RF: Validation, Writing – review & editing. FC: Validation, Writing – review & editing. GL: Validation, Writing – review & editing. AS: Validation, Writing – review & editing. CG: Validation, Writing – review & editing. MC: Validation, Writing – review & editing. RG: Validation, Writing – review & editing. EG: Validation, Writing – review & editing. FA: Validation, Writing – review & editing. RC: Validation, Writing – review & editing. FI: Validation, Writing – review & editing. MS: Writing – review & editing, Writing – original draft.

## References

[1] Serhal L Lwin MN Holroyd C Edwards CJ. Rheumatoid arthritis in the elderly: characteristics and treatment considerations. Autoimmun Rev. (2020) 19:102528. doi: 10.1016/j.autrev.2020.102528, PMID: 32234572

[2] Aletaha D Neogi T Silman AJ Funovits J Felson DT Bingham CO. 2010 rheumatoid arthritis classification criteria: an American College of Rheumatology/European league against rheumatism collaborative initiative. Arthritis Rheum. (2010) 62:2569–81. doi: 10.1002/art.27584, PMID: 20872595

[3] Rasch EK Hirsch R Paulose-Ram R Hochberg MC. Prevalence of rheumatoid arthritis in persons 60 years of age and older in the United States: effect of different methods of case classification. Arthritis Rheum. (2003) 48:917–26. doi: 10.1002/art.10897, PMID: 12687533

[4] Boots AMH Maier AB Stinissen P Masson P Lories RJ De Keyser F. The influence of ageing on the development and management of rheumatoid arthritis. Nat Rev Rheumatol. (2013) 9:604–13. doi: 10.1038/nrrheum.2013.92, PMID: 23774902

[5] Nawrot J Boonen A Peeters R Starmans M van Onna M. Rheumatologists’ views and experiences in managing rheumatoid arthritis in elderly patients: a qualitative study. J Rheumatol. (2018) 45:590–4. doi: 10.3899/jrheum.170773, PMID: 29449497

[6] Konrat C Boutron I Trinquart L Auleley GR Ricordeau P Ravaud P. Underrepresentation of elderly people in randomised controlled trials. The example of trials of 4 widely prescribed drugs. PLoS One. (2012) 7:e33559. doi: 10.1371/journal.pone.0033559, PMID: 22479411 PMC3316581

[7] Meisters R Putrik P Ramiro S Hifinger M Keszei AP van Eijk-Hustings Y. EULAR/eumusc.net standards of care for rheumatoid arthritis: cross-sectional analyses of importance, level of implementation and care gaps experienced by patients and rheumatologists across 35 European countries. Ann Rheum Dis. (2020) 79:1423–31. doi: 10.1136/annrheumdis-2020-217520, PMID: 32873554

[8] Sebastiani M Anelli MG Atzeni F Bazzani C Farina I Fedele AL. Efficacy and safety of rituximab with and without methotrexate in the treatment of rheumatoid arthritis patients: results from the GISEA register. Joint Bone Spine. (2014) 81:508–12. doi: 10.1016/j.jbspin.2014.06.011, PMID: 25082646

[9] Smolen JS Landewé RBM Bergstra SA Kerschbaumer A Sepriano A Aletaha D. EULAR recommendations for the management of rheumatoid arthritis with synthetic and biological disease-modifying antirheumatic drugs: 2022 update. Ann Rheum Dis. (2023) 82:3–18. doi: 10.1136/ard-2022-223356, PMID: 36357155

[10] Chan W. Statistical methods in medical research. Model Assist Stat Appl. (2013) 8:83–4. doi: 10.3233/MAS-130255

[11] Ohta R Sano C. Differentiating between seronegative elderly-onset rheumatoid arthritis and polymyalgia rheumatica: a qualitative synthesis of narrative reviews. Int J Environ Res Public Health. (2023) 20:1789. doi: 10.3390/ijerph20031789, PMID: 36767155 PMC9914621

[12] Wu J Yang F Ma X Lin J Chen W. Elderly-onset rheumatoid arthritis vs. polymyalgia rheumatica: differences in pathogenesis. Front Med (Lausanne). (2023) 9:1083879. doi: 10.3389/fmed.2022.1083879, PMID: 36714116 PMC9879490

[13] Fraenkel L Rabidou N Dhar R. Are rheumatologists’ treatment decisions influenced by patients’ age? Rheumatology. (2006) 45:1555–7. doi: 10.1093/rheumatology/kel144, PMID: 16690762 PMC1660557

[14] Sugihara T. Treatment strategies for elderly-onset rheumatoid arthritis in the new era. Mod Rheumatol. (2022) 32:493–9. doi: 10.1093/mr/roab087, PMID: 34791359

[15] Mathieu S Pereira B Saraux A Richez C Combe B Soubrier M. Disease-modifying drug retention rate according to patient age in patients with early rheumatoid arthritis: analysis of the ESPOIR cohort. Rheumatol Int. (2021) 41:879–85. doi: 10.1007/s00296-020-04770-7, PMID: 33433729

[16] Ebina K Hashimoto M Yamamoto W Hirano T Hara R Katayama M. Drug tolerability and reasons for discontinuation of seven biologics in elderly patients with rheumatoid arthritis -the ANSWER cohort study. PLoS One. (2019) 14:e0216624. doi: 10.1371/journal.pone.0216624, PMID: 31067271 PMC6505948

[17] Arnold J Winthrop K Emery P. COVID-19 vaccination and antirheumatic therapy. Rheumatology. (2021) 60:3496–502. doi: 10.1093/rheumatology/keab223, PMID: 33710296 PMC7989162

[18] Naveen R Parodis I Joshi M Sen P Lindblom J Agarwal V. COVID-19 vaccination in autoimmune diseases (COVAD) study: vaccine safety and tolerance in rheumatoid arthritis. Rheumatology. (2023) 62:2366–76. doi: 10.1093/rheumatology/keac624, PMID: 36315075

[19] Klebanoff SD Rodda LB Morishima C Wener MH Yuzefpolskiy Y Bettelli E. Diminished responses to mRNA-based SARS-CoV-2 vaccines in individuals with rheumatoid arthritis on immune-modifying therapies. JCI Insight. (2023) 8:168663. doi: 10.1172/jci.insight.168663 PMC10445680 37338983

[20] Sebastiani M Venerito V Bugatti S Bazzani C Biggioggero M Petricca L. Retention rate of a second line with a biologic DMARD after failure of a first-line therapy with abatacept, tocilizumab, or rituximab: results from the Italian GISEA registry. Clin Rheumatol. (2021) 40:4039–47. doi: 10.1007/s10067-021-05734-3, PMID: 33881676

[21] Iannone F Gremese E Atzeni F Biasi D Botsios C Cipriani P. Longterm retention of tumor necrosis factor-α inhibitor therapy in a large Italian cohort of patients with rheumatoid arthritis from the GISEA registry: an appraisal of predictors. J Rheumatol. (2012) 39:1179–84. doi: 10.3899/jrheum.111125, PMID: 22467933

[22] Temmoku J Migita K Yoshida S Matsumoto H Fujita Y Matsuoka N. Real-world comparative effectiveness of bDMARDs and JAK inhibitors in elderly patients with rheumatoid arthritis. Medicine. (2022) 101:e31161. doi: 10.1097/MD.0000000000031161, PMID: 36281115 PMC9592439

[23] Busquets N Tomero E Descalzo MÁ Ponce A Ortiz-Santamaría V Surís X. Age at treatment predicts reason for discontinuation of TNF antagonists: data from the BIOBADASER 2.0 registry. Rheumatology. (2011) 50:1999–2004. doi: 10.1093/rheumatology/ker281, PMID: 21856725

[24] Bechman K Oke A Yates M Norton S Dennison E Cope AP. Is background methotrexate advantageous in extending TNF inhibitor drug survival in elderly patients with rheumatoid arthritis? An analysis of the British Society for Rheumatology biologics register. Rheumatology. (2020) 59:2563–71. doi: 10.1093/rheumatology/kez671, PMID: 31998962 PMC7449803

[25] Frasca D Diaz A Romero M Landin AM Blomberg BB. Age effects on B cells and humoral immunity in humans. Ageing Res Rev. (2011) 10:330–5. doi: 10.1016/j.arr.2010.08.004, PMID: 20728581 PMC3040253

[26] Specker C Aringer M Burmester GR Killy B Hofmann MW Kellner H. The safety and effectiveness of tocilizumab in elderly patients with rheumatoid arthritis and in patients with comorbidities associated with age. Clin Exp Rheumatol. (2021) 40:1657–65. doi: 10.55563/clinexprheumatol/f7ff6q, PMID: 34874836

[27] Sato S Matsumoto H Temmoku J Fujita Y Matsuoka N Yashiro-Furuya M. Sustained long-term retention rates of abatacept in combination with conventional synthetic disease-modifying antirheumatic drugs in elderly patients with rheumatoid arthritis. Medicina (B Aires). (2021) 57:914. doi: 10.3390/medicina57090914, PMID: 34577837 PMC8469009

[28] Charles-Schoeman C Buch MH Dougados M Bhatt DL Giles JT Ytterberg SR. Risk of major adverse cardiovascular events with tofacitinib versus tumour necrosis factor inhibitors in patients with rheumatoid arthritis with or without a history of atherosclerotic cardiovascular disease: a post hoc analysis from ORAL surveillance. Ann Rheum Dis. (2023) 82:119–29. doi: 10.1136/ard-2022-222259, PMID: 36137735 PMC9811099

[29] Temmoku J Miyata M Suzuki E Sumichika Y Saito K Yoshida S. Drug retention rates and the safety of Janus kinase inhibitors in elderly patients with rheumatoid arthritis. J Clin Med. (2023) 12:4585. doi: 10.3390/jcm12144585, PMID: 37510700 PMC10380728

[30] Gialouri CG Pappa M Evangelatos G Nikiphorou E Fragoulis GE. Effect of body mass index on treatment response of biologic/targeted-synthetic DMARDs in patients with rheumatoid arthritis, psoriatic arthritis or axial spondyloarthritis. A systematic review. Autoimmun Rev. (2023) 22:103357. doi: 10.1016/j.autrev.2023.103357 37150489

[31] Novella-Navarro M Genre F Hernández-Breijo B Remuzgo-Martínez S Martínez-Feito A Peiteado D. Obesity and response to biological therapy in rheumatoid arthritis: the role of body mass index and adipose tissue cytokines. Clin Exp Rheumatol. (2021) 40:1726–32. doi: 10.55563/clinexprheumatol/a9gskx, PMID: 35084302

[32] Moschou D Krikelis M Georgakopoulos C Mole E Chronopoulos E Tournis S. Sarcopenia in rheumatoid arthritis. A narrative review. J Frailty Sarcopenia Falls. (2023) 8:44–52. doi: 10.22540/JFSF-08-044, PMID: 36873824 PMC9975974

[33] Ytterberg SR Bhatt DL Mikuls TR Koch GG Fleischmann R Rivas JL. Cardiovascular and cancer risk with tofacitinib in rheumatoid arthritis. N Engl J Med. (2022) 386:316–26. doi: 10.1056/NEJMoa2109927 35081280

